# Bilateral Sever’s Disease in Identical Twin Sisters: A Case Report

**DOI:** 10.7759/cureus.83273

**Published:** 2025-04-30

**Authors:** Ayan Baur, Anoovab Saha, Raktim Swarnakar, Pankaj K Mandal, Soumyadipta Ghosh

**Affiliations:** 1 Physical Medicine and Rehabilitation, Radha Gobinda (RG) Kar Medical College and Hospital, Kolkata, IND; 2 Physical Medicine and Rehabilitation, National Cancer Institute (NCI) Jhajjar Campus, All India Institute of Medical Sciences (AIIMS), New Delhi, IND

**Keywords:** calcaneal pain, heel and sole pain, identical twins, overuse injury, physical medicine and rehabilitation (pm&r), sever's apophysitis, sever’s disease

## Abstract

Sever’s disease, or calcaneal apophysitis, is the primary cause of heel pain in pediatric patients. However, its simultaneous occurrence in monozygotic twin siblings is rarely documented. It is commonly seen in children aged between 8 and 15 years with skeletal immaturity. This case report presents 10-year-old Indian monozygotic twin sisters from a lower socioeconomic background in a rural area who simultaneously developed bilateral Sever’s disease. Both twins reported a gradual onset of bilateral heel pain over seven months, which was exacerbated by walking and physical activity.

On examination, both twins exhibited a moderate build, weighing 26 kg and 27 kg, respectively. They displayed bilateral hallux varus deformities, and the calcaneal squeeze test was positive bilaterally, which was sufficient to establish the diagnosis. Radiological evaluation, including digital X-rays of the bilateral feet in anteroposterior and lateral views, confirmed the presence of calcaneal apophysitis. Given the identical clinical presentation and radiographic findings in both twins, this case raises the possibility of an underlying genetic predisposition or shared biomechanical risk factors.

This report highlights the need for further research into potential hereditary influences contributing to Sever’s disease and suggests that genetic, environmental, and mechanical stressors may synergistically contribute to its pathogenesis.

## Introduction

Sever’s disease, or calcaneal apophysitis, is a common cause of heel pain in children and adolescents, particularly those aged 5 to 14 years, with peak incidence occurring between 9 and 12 years [1]. It is classified as an overuse injury, resulting from repetitive microtrauma at the secondary ossification center (apophysis, a normal secondary ossification center located in the non-weight-bearing part of the bone that eventually fuses with it over time) of the calcaneus [2]. This condition arises due to excessive tensile stress from the Achilles tendon at its insertion site, leading to inflammation and irritation of the calcaneal apophysis [3]. The apophyseal region remains cartilaginous until approximately 12 to 15 years of age, rendering it particularly vulnerable to stress-induced inflammation.

Sever’s disease is frequently observed in physically active children involved in sports or activities that require frequent running and jumping [4]. Symptoms typically include activity-related posterior heel pain, localized tenderness at the calcaneal apophysis, and pain aggravated by physical activity, with relief upon rest, offloading techniques, and ice application [5]. Diagnosis is primarily clinical, supported by a positive calcaneal squeeze test and imaging when necessary [2]. The condition is self-limiting and usually resolves once skeletal maturity is reached [6].

Although previous studies have identified mechanical stress and environmental factors as primary contributors, no definitive genetic basis has been established. The occurrence of Sever’s disease in identical twins, as noted in this case, suggests a possible hereditary component that warrants further investigation.

## Case presentation

A pair of 10-year-old monozygotic twin girls from a rural area and a lower socioeconomic background presented to the Physical Medicine and Rehabilitation outpatient department with bilateral heel pain persisting for the past seven months. The pain began insidiously, gradually worsened, and was exacerbated by weight-bearing activities such as walking and playing. There was no pain at rest and no pain during the first step in the morning. There was no history of trauma, fever, recent illness, or prior musculoskeletal complaints. Their activity level was age-appropriate, and they were not involved in any specific sports. Dietary history did not suggest any nutritional deficiencies. Both girls alternated between using slippers and shoes. The twins' mother reported that both were delivered via cesarean section and had achieved all developmental milestones appropriately, with no prior history of systemic illness or joint deformities. There was no family history of similar conditions.

**Figure 1:**
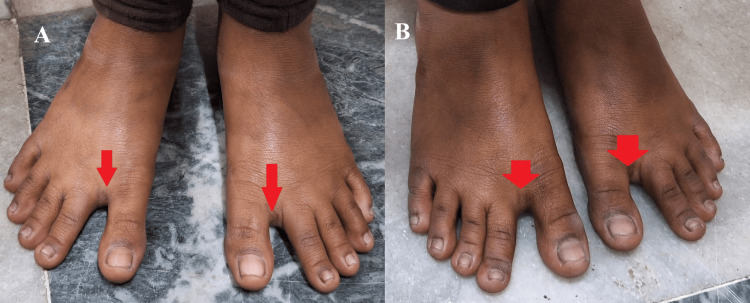
Bilateral hallux varus deformity observed in both twins. A) Hallux deformity (red arrow) in Twin 1; B) Hallux deformity (red arrow) in Twin 2.

On clinical examination, both girls exhibited a moderate build, weighing 26 kg and 27 kg respectively, with a height of 55 inches each. Bilateral hallux varus deformities were noted (Figure 1, panels A and B).

The calcaneal squeeze test (gently squeezing the heel from both sides, applying pressure to the medial and lateral surfaces of the calcaneus) was positive bilaterally, eliciting mild-to-moderate pain at the calcaneal apophysis. There was no restriction in the range of motion at the ankle, metatarsophalangeal, or interphalangeal joints. No signs of infection, erythema, or systemic inflammatory conditions were observed. No other joint deformities were noted. No tightness of the gastrosoleus or plantar fascia was found. The medial arches of both feet were mildly reduced. Gait was normal but mildly slowed due to pain. 

**Figure 2:**
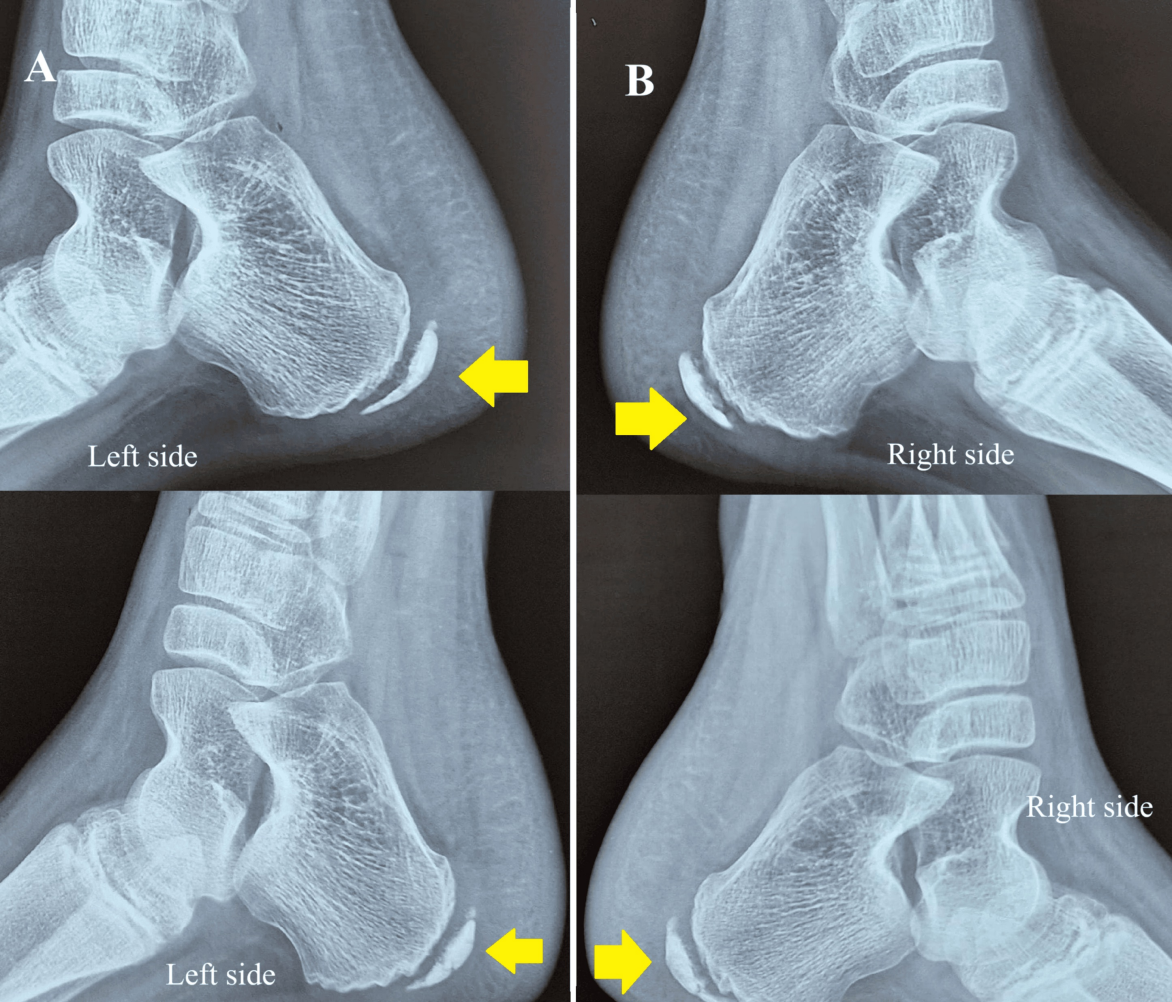
X-rays of the right and left calcaneus in both twins (lateral view). A) Lateral view X-ray of the left calcaneus in Twin 1 (top image) and Twin 2 (bottom image), showing increased density and fragmentation of the calcaneal apophysis (yellow arrow); B) Lateral view X-ray of the right calcaneus in Twin 1 (top image) and Twin 2 (bottom image), also showing increased density and fragmentation of the calcaneal apophysis (yellow arrow).

Radiological evaluation included digital X-rays of both feet in anteroposterior and lateral views. The X-rays showed increased density and fragmentation of the calcaneal apophysis in both twins (Figure 2, panels A and B).

Although the diagnosis of calcaneal apophysitis is primarily clinical, the X-rays showed no signs of fractures, osteomyelitis, or other pathological findings [7]. Routine blood investigations, including complete blood count (with total leukocyte count), erythrocyte sedimentation rate, C-reactive protein, liver function tests, kidney function tests, serum alkaline phosphatase, serum albumin, and total protein were all within normal limits.

Management was carried out according to evidence-based guidelines [8]. Pain was managed with paracetamol 500 mg (as needed) and topical non-steroidal anti-inflammatory drug (diclofenac 1% gel) applied twice daily for 7 days. Ice fomentation, bilateral silicone heel cushions/cups, and stretching exercises for the gastrocnemius and soleus muscles were also recommended. Activity pacing was advised. The simultaneous and identical presentation of Sever’s disease in both twins raises significant questions regarding its pathophysiology and potential hereditary and environmental influences.

## Discussion

Sever's disease is traditionally considered a mechanical overuse injury, with risk factors including excessive physical activity, high-impact sports, tight calf muscles, flat feet, or a high-arched foot structure [2,9]. However, its simultaneous occurrence in identical twins presents a unique scenario, suggesting a potential underlying genetic predisposition. While previous studies have not established a direct genetic link, twin studies in other musculoskeletal disorders, such as Achilles tendinopathy and osteochondrosis, suggest that hereditary factors may contribute to susceptibility.

Approximately 60% of patients with Sever's disease experience bilateral heel pain. It is more common in males, with a frequency 2 to 3 times higher than in females. The presence of bilateral hallux varus deformities in both twins further supports the idea of shared biomechanical or genetic influences [4]. Identical twins often exhibit similar gait mechanics, foot morphology, and neuromuscular development, which could lead to equal stress distribution on their Achilles tendons, predisposing them to Sever's disease [5]. Additionally, since the twins are from a rural setting, it is likely they engaged in similar physical activities, reinforcing the possibility of common environmental triggers.

Nutritional factors may also play a role in delayed ossification of the calcaneal apophysis, potentially prolonging the risk window for Sever's disease. Given their lower socioeconomic status, dietary deficiencies could further contribute to the condition.

Diagnosis of Sever's disease is typically clinical, and most laboratory investigations are within normal limits [4]. The lack of a genetic study is a limitation of this case report; however, interestingly, no genetic studies on Sever's disease have been reported in the literature to date. Furthermore, the prognosis for Sever's disease is generally good, as most cases are self-limiting and resolve with skeletal maturation and apophyseal closure [10].

This case report raises the question of whether Sever's disease should be investigated for a potential genetic component, particularly in cases occurring in multiple family members. Further research, including genetic studies and twin cohort analyses, may help determine whether certain genetic markers predispose individuals to calcaneal apophysitis. Additionally, longitudinal studies on familial clustering of Sever's disease could provide further insights into its hereditary aspects.

## Conclusions

This case represents a rare instance of identical twin sisters with Sever’s disease, suggesting a possible genetic predisposition. While current literature attributes Sever’s disease primarily to mechanical stress and environmental factors, this report indicates that hereditary influences may also play a role. The twins’ identical presentation, radiographic findings, and clinical course suggest an interaction between genetic, biomechanical, and environmental factors.

Sever’s disease is a self-limiting condition, and early diagnosis with appropriate conservative management typically leads to improvement. Although this case raises interesting questions about potential genetic factors, it primarily underscores the need for further research rather than providing conclusive evidence. Nonetheless, it highlights the importance of conducting additional genetic and epidemiological studies to explore the potential hereditary component of Sever’s disease. Investigating familial patterns and genetic predisposition could enhance our understanding of its pathogenesis and support the development of preventive strategies for at-risk populations.
